# Breast Adipocyte Co-culture Increases the Expression of Pro-angiogenic Factors in Macrophages

**DOI:** 10.3389/fonc.2020.00454

**Published:** 2020-04-07

**Authors:** Nalini V. S. Yadav, Arthur Barcikowski, Yuko Uehana, Aaron T. Jacobs, Linda Connelly

**Affiliations:** ^1^Department of Pharmaceutical Sciences, Daniel K. Inouye College of Pharmacy, University of Hawaii at Hilo, Hilo, HI, United States; ^2^School of Medicine, California University of Science and Medicine, San Bernardino, CA, United States

**Keywords:** obesity, breast, adipocytes, macrophages, vascular endothelial growth factor (VEGF)

## Abstract

Obese individuals with breast cancer have a poorer prognosis and higher risk of metastatic disease vs. non-obese patients. Adipose tissue in obese individuals is characterized by an enhanced macrophage infiltration, creating a microenvironment that favors tumor progression. Here, we demonstrate a role for adipocyte-macrophage interactions in the regulation of angiogenesis. Co-culture of THP-1 macrophages with human breast adipocytes led to increased expression of the pro-angiogenic growth factor, vascular endothelial growth factor A (VEGFA). Several adipocyte-derived proteins including leptin, insulin, IL-6, and TNF-α were each capable of increasing VEGFA expression in THP-1 macrophages, identifying these as possible mediators of the changes that were observed with co-culture. Furthermore, analysis of THP-1 culture media by antibody array revealed that THP-1 secrete several other pro-angiogenic signals in response to adipocyte co-culture, including interleukin 8 (IL-8), matrix metalloproteinase 9 (MMP9), pentraxin 3 (PTX3), and serpin E1 (plasminogen activator inhibitor 1, PAI1) after co-culture with human adipocytes. We used an *in vitro* endothelial tube formation assay with human vascular endothelial cells to evaluate the effects of THP-1 culture media on angiogenesis. Here, culture media from THP-1 cells previously exposed to human adipocytes stimulated endothelial tube formation more significantly than THP-1 cells cultured alone. In summary, we find that adipocyte co-culture stimulates the expression of pro-angiogenic mediators in macrophages and has pro-angiogenic effects *in vitro*, thus representing a possible mechanism for the enhanced risk of breast cancer progression in obese individuals.

## Introduction

Approximately 40% of the female population of the United States is obese, which is defined as having a body mass index (BMI) ≥ 30.0 kg/m^2^ (1, 2). Globally, the mean BMI value for women has increased steadily by 2.3 kg/m^2^ over the past four decades (3). Being obese is a well-established risk factor for a number of chronic disease states, including heart disease, stroke, type 2 diabetes, and several types of cancer, including breast cancer (4–6). Epidemiological studies have demonstrated that obese women who are postmenopausal are at greater risk for developing breast cancer when compared with non-obese women (7, 8). For women of all age groups diagnosed with breast cancer, obesity correlates strongly with disease progression, tumor burden, and advanced tumor stage (9–11). Moreover, obese women have worse clinical outlook than their non-obese counterparts (12). Accordingly, among 2,755 U.S. women with breast cancer, those with a BMI of 30.0–34.9 mg/kg^2^ had a 1.63 relative risk of cancer-related death when compared to patients with normal BMI of 18.5–24.99 mg/kg^2^ (13).

Obesity is characterized by the expansion of adipose tissue in the body, which is composed of various cell types including adipocytes, endothelial cells, fibroblasts, and immune cells, including macrophages. The cellular composition and gene expression profiles of adipose tissue from obese individuals is different than individuals with a normal BMI. In particular, obese adipose tissue shows a high degree of macrophage infiltration. In visceral adipose tissue of non-obese individuals, macrophages account for 10–15% of stromal vascular cells but are increased to 40–50% in obese individuals (14, 15). A high level of macrophage infiltration has also been observed in obese breast tissue and is accompanied by an increase in the expression of pro-inflammatory genes (16).

Within obese breast tissue, macrophages have established roles in both tumor growth and metastasis. Imaging of breast tumors has demonstrated that invasion of breast cancer cells occurs in association with tumor-associated macrophages (TAMs), supporting the notion that TAMs promote both tumor invasion and metastasis (17). TAMs cluster within areas exhibiting a high level of angiogenic activity and are therefore suspected to promote vascularization (18, 19). Characterization of breast tissue macrophages showed that macrophages from obese women were similar to TAMs (20). A recent study found that fatty breast tissue combined with a high level of TAMs increased the risk of breast cancer recurrence and mortality (21).

In order to learn more about the cellular interactions in the obese breast tissue microenvironment we have investigated the role of breast adipocytes in stimulating a tumor-promoting macrophage phenotype. Our study reveals that breast adipocytes release soluble factors which alter the macrophage phenotype and lead to the up-regulation of pro-angiogenic signaling, which is a critical step in tumor growth and progression to metastatic disease.

## Materials and Methods

### Cell Culture and Reagents

Human monocytic cell line THP-1, murine fibroblast-like cell line 3T3-L1, and murine macrophage-like cell line J774A.1 were purchased from the American Type Culture Collection (ATCC, Manassas, VA, USA). Primary human breast preadipocytes isolated from patients with BMI>30 were commercially obtained (ZenBio, Research Triangle Park, NC, USA) and expanded in Preadipocyte Medium (PM-1). All cells were maintained at 37°C in humidified incubator containing 5% CO_2_. THP-1 were differentiated with 25 ng/mL phorbol 12-myristate 13-acetate (PMA) for 24 h. Primary breast preadipocytes were differentiated in Adipocyte Differentiation Medium (DM-2) for 15 days, after which media was changed to Adipocyte Maintenance Medium (AM-1) and cells were allowed to rest for 1 day. Cells were examined for lipid droplet accumulation and differentiation was confirmed by AdipoRed assay (Lonza, Basel, Switzerland) as well as expression of leptin by PCR. Murine 3T3-L1 cells were differentiated to an adipocyte phenotype by incubation in DMEM supplemented with 5 μg/mL insulin, 0.5 mM isobutylmethylxanthine, and 0.25 μM dexamethasone for 48 h. Media was then replaced every other day with DMEM supplemented with 5 μg/mL insulin only for a total of 10 additional days. Cells were visually checked for lipid droplet accumulation and differentiation was confirmed by AdipoRed assay.

### Co-culture Experiments

Differentiated primary human adipocytes were prepared for co-culture by changing media to fresh AM-1. THP-1 were seeded on Transwell inserts (Corning, Corning, NY, USA) with a 0.4 μm pore size polycarbonate permeable membrane at a density of 5 × 10^5^ cells per 4.67 cm^2^ and differentiated with PMA. Inserts were then placed in 6-well plates that either contained or did not contain primary human adipocytes. Murine cell culture experiments were performed in a similar manner. 3T3-L1 adipocytes were prepared for co-culture by changing media to fresh DMEM + insulin. Murine J774A.1 cells were seeded on Transwell inserts, then placed in 6-wells plates that either contained or did not contain 3T3-L1 adipocytes. Macrophage RNA was collected following 8 h of co-culture with adipocytes. For protein samples, Transwell inserts were removed after 8 h of co-culture and placed in fresh culture media without adipocytes for an additional 16 h. Similarly, for ELISA, Luminex, and angiogenesis studies, cell culture supernatant was collected after an additional 16 h in fresh culture media without adipocytes.

### RNA Isolation and Quantitative Reverse Transcription PCR (qRT-PCR)

To extract total RNA, macrophages were scraped, pelleted by centrifugation and resuspended in TRIzol reagent (Invitrogen, Carlsbad CA, USA). Chloroform was added and samples allowed to incubate at 25°C for 5 min then centrifuged. The aqueous layer was transferred to a fresh tube to which an equal volume of 70% ethanol was added. RNA was subsequently purified using the RNeasy Mini Kit (Qiagen, Valencia, CA USA). DNA contamination was removed with the DNA-free kit (Invitrogen) then RNA samples were quantified by UV absorbance at 260 nm. RNA was reverse-transcribed using the iScript cDNA Synthesis Kit (BioRad, Hercules, CA, USA) using one cycle of 25°C for 5 min, 42°C for 30 min, and 85°C for 5 min. Real-time PCR was performed using iQ SYBR Green Supermix (BioRad) with 400 nM primers (IDT, Coralville, IA, USA) in a CFX96 quantitative PCR detection system (BioRad). Relative fluorescent units (RFU) were plotted against cycle number using the BioRad CFX Manager software (BioRad). RFU is a quantitative measurement dependent on the amount of PCR product. The threshold cycle (Ct) values for the target genes were normalized to glyceraldehyde 3-phosphate dehydrogenase (GAPDH) or 18S ribosomal subunit (18S) gene expression, obtaining ΔCt = Ct (GAPDH or 18S)—Ct (target). Each cDNA sample was amplified in replicates of 3 for each PCR target and control. RT negative control was no reverse transcriptase and quantitative PCR negative control was no template control in triplicate for each target. Primer sequences are shown in Table 1.

**Table 1:** PCR primer sequences.

**Gene**	**Species**	**Forward primer sequence (5′–3′)**	**Reverse primer sequence (5′–3′)**
*GAPDH*	Human	TCGACAGTCAGCCGCATCTTCTTT	ACCAAATCCGTTTCCGACCTT
*RNA18SN5 (18S)*	Human	GCCCGAGCCGCCTGGATACC	TCACCTCTAGCGGCGCAATACG
*Gapdh*	Mouse	TGAGGACCAGGTTGTCTCCT	CCCTGTTGCTGTAGCCGTAT
*VEGFA*	Human	ACACATTGTTGGAAGAAGCAGCCC	AGGAAGGTCAACCACTCACACACA
*Vegfa*	Mouse	AGGCTGCTGTAACGATGAAG	TCTCCTATGTGCTGGCTTTG
*PTGS2 (COX2)*	Human	ATCTACCCTCCTCAAGTCCC	AACAACTGCTCATCACCCC
*MRC1 (mannose receptor)*	Human	TGTCCAGAAGGTGACTGTTTAG	CTAACAGCGCAGCAAGAAATC
*IL6*	Human	GCCTGCATTAGGAGGTCTTT	CCTGACACCAGCAAAGGATAA
*IL10*	Human	CAACCTGCCTAACATGCTTC	CCAGGTAACCCTTAAAGTCCTC

### VEGFA ELISA and Luminex Multiplex

After 8 h co-culture with adipocytes, Transwell inserts containing macrophages were transferred to fresh culture media without adipocytes for an additional 16 h. THP-1 cell culture supernatants were analyzed by Ocean Ridge Biosciences (Palm Beach Gardens, FL, USA) using a Luminex xMAP Multiplex for VEGFA protein concentrations. Secreted VEGFA protein concentrations were assessed in murine cell culture supernatants using the Murine VEGFA Standard ELISA Development Kit (PeproTech, Rocky Hill, NJ, USA). Each standard and sample were analyzed in triplicate for each experiment.

### Endothelial Tube Formation Assay

Human umbilical vein endothelial cells (HUVEC) were purchased from Life Technologies (Carlsbad, CA, USA) and maintained in Medium 200 PRF supplemented with Large Vessel Endothelial Supplement and 50 μg/mL antibiotic/antimycotic solution at 37°C in humidified atmosphere containing 5% CO_2_. Cells were harvested and resuspended in either serum free medium, full serum medium, or sample-conditioned medium from macrophages either exposed to adipocytes or not. Cells were added to cell culture vessels coated with Geltrex (Invitrogen, Carlsbad, CA, USA) and allowed to incubate for 14–16 h, then imaged. Images were collected randomly and blinded using a Zeiss Axiovert inverted microscope with a Canon EOS Rebel T3i camera attached using a 2.5X objective. Photographs were then auto leveled and resized, then analyzed in ImageJ (www.imagej.nih.gov/ij/) using an Angiogenesis Analyzer tool developed by Gilles Carpentier (www.image.bio.methods.free.fr/ImageJ/?Angiogenesis-Analyzer-for-ImageJ) (22).

### Human Angiogenesis Array

Conditioned media from differentiated THP-1 either cultured alone or cultured with differentiated primary human breast adipocytes were analyzed using the Proteome Profiler Human Angiogenesis Array (R&D Systems, Minneapolis, MN, USA). Conditioned media from each sample was run on an individual array membrane, which was scanned using a Bio-Rad ChemiDoc XRS+ gel documentation system and measured using Bio-Rad Image Lab software. Intensities were normalized to reference spots on each membrane.

### Statistical Analyses

Statistical analyses were performed using Microsoft Excel unless otherwise noted. Data plotted graphically included vertical bars representing standard error. The unpaired Student's *t*-test was used to assess differences in relative levels of mRNA and protein. HUVEC tube formation assay parameters were analyzed using a two-way Analysis of Variance (ANOVA) model, with factors for experiment (*n* = 4) and treatment (macrophages cultured alone vs. macrophages co-cultured with adipocytes). An additive main effect model, exclusive of an interaction, was implemented and analyses were performed using SAS version 9.3. A probability (*P*) value of <0.05 was taken as an appropriate level of significance.

## Results

### Establishing a Human Breast Adipocyte and Macrophage Co-culture System

**Figure 1:**
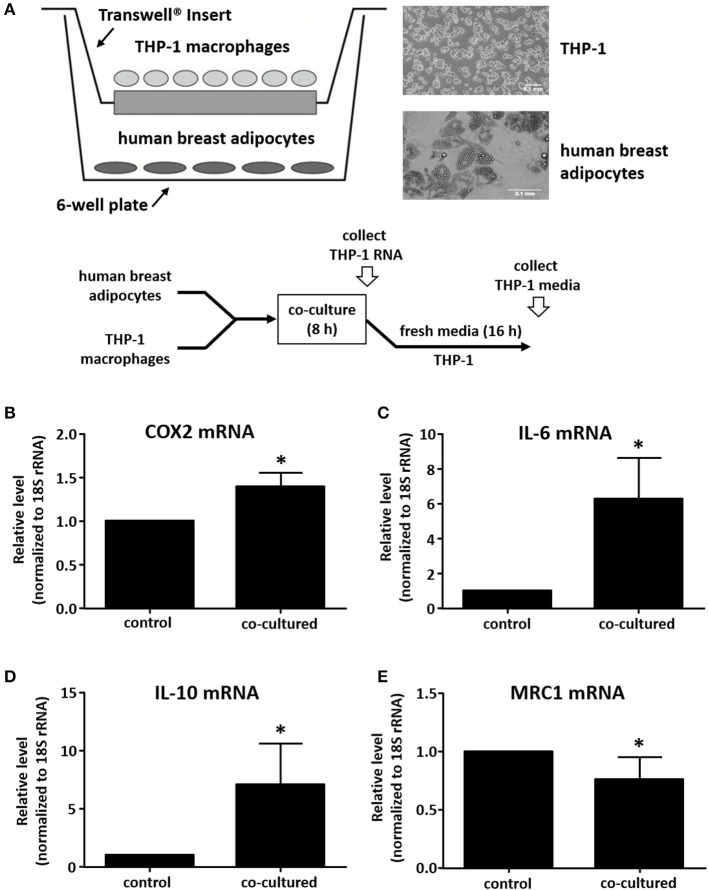
Human adipocyte-macrophage co-culture system and phenotype determination of adipocyte-exposed macrophages. **(A)** Diagram showing co-culture system where differentiated primary human breast adipocytes were cultured in the lower chamber, PMA-differentiated THP-1 were cultured on a Transwell insert, and co-culture was performed for 8 h prior to RNA collection. THP-1 mRNA levels were analyzed by real time qPCR to determine expression of COX2 **(B)**, IL-6 **(C)**, IL-10 **(D)**, or MRC1 **(E)**; values were normalized to 18S RNA, *n* = 4, ^*^p < 0.05, statistical analyses were performed using standard deviation and the unpaired Student's *t*-test.

We utilized a co-culture system to examine the impact of adipocytes on gene expression in macrophages. Human breast preadipocytes were differentiated into mature adipocytes as described in Materials and Methods. THP-1 monocytic cells were seeded onto Transwell inserts, differentiated into macrophages with PMA, then subsequently co-cultured with differentiated adipocytes for 8 h after which total RNA was extracted from the THP-1 cells (Figure 1A). As a negative control, macrophages were seeded into a Transwell insert and incubated in media only, without adipocytes in the lower culture chamber. We performed qPCR to examine the effect of co-culture on macrophage phenotype. Samples were analyzed for the expression of markers of an M1 (classically activated) phenotype, which represents a pro-inflammatory immune response, and markers of an M2 (alternatively activated) phenotype, which represents an anti-inflammatory immune response (23–26). Two established M1 phenotype markers were selected: COX2 and IL-6. Also, two M2 phenotype markers were examined: IL-10 and MRC1. Values were normalized to 18S rRNA levels and expressed relative to negative controls (THP-1 cultured without adipocytes). The data show that adipocyte-exposed THP-1 macrophages acquired a mixed M1/M2 phenotype, with increased expression of COX2, IL-6, and IL-10 and decreased expression of MRC1, relative to THP-1 macrophages cultured alone (Figures 1B–E).

### Exposure to Adipocytes Stimulates Macrophage VEGFA Expression

**Figure 2:**
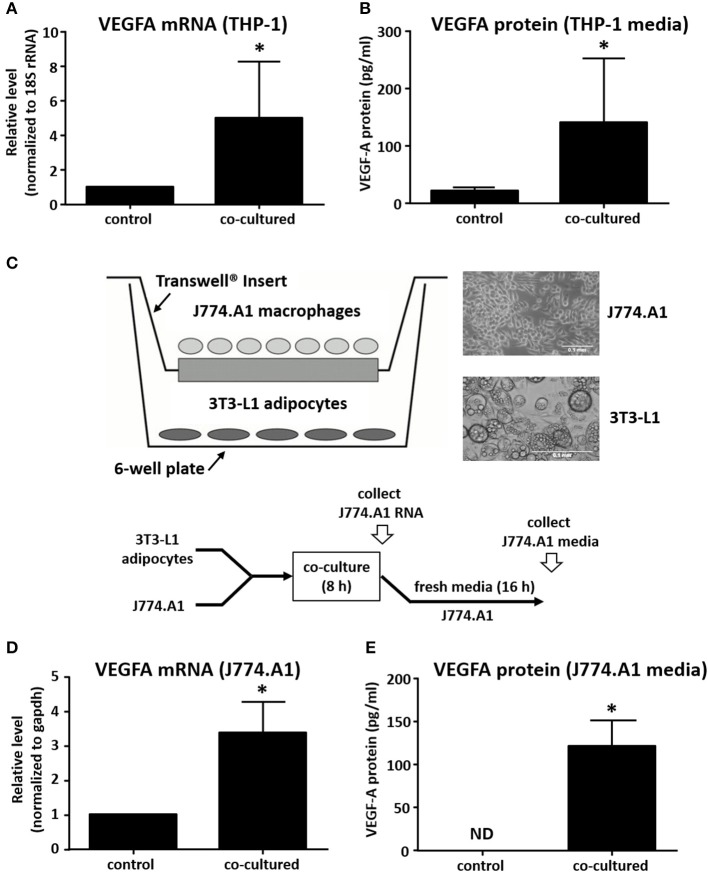
Co-culture with adipocytes stimulates expression of VEGF in macrophages. **(A)** Differentiated primary human breast adipocytes were co-cultured with PMA-differentiated THP-1 cells using a Transwell system for 8 h before RNA was collected, reverse transcribed, and qPCR performed with 18S and VEGFA primers. **(B)** THP-1 were co-cultured with breast adipocytes as above for 8 h then changed to fresh media for an additional 16 h, then supernatant was collected and analyzed for secreted VEGFA protein. **(C)** Diagram showing co-culture system where murine adipocytes were cultured in the lower chamber and murine macrophages were cultured on a Transwell insert, co-culture was performed for 8 h. **(D)** Macrophage RNA was collected after 8 h of co-culture, reverse transcribed, and qPCR performed with GAPDH and VEGFA primers. **(E)** J774A.1 murine macrophages were co-cultured with differentiated 3T3-L1 murine adipocytes as above for 8 h then changed to new media for 16 additional h before supernatant was collected and analyzed for secreted proteins (*n* = 4, ^*^p < 0.05); ND, not detected; statistical analyses were performed using standard deviation and the unpaired Student's *t*-test.

We next investigated whether macrophage-derived signals with potential roles in breast cancer angiogenesis were influenced by adipocyte co-culture. This is of particular interest because the recruitment of blood vessels to primary tumors has a critical role in tumor invasion and metastasis. VEGFA has a mitogenic effect on vascular endothelial cells, stimulating endothelial tube formation and neovascularization of the tumor (27). Macrophages in the breast tumor microenvironment have been previously described as secreting VEGFA, but the role of adipocytes in regulating this process is not known (28). We observed an approximate 5-fold increase in macrophage expression of VEGFA mRNA after co-culture with adipocytes for 8 h (Figure 2A). In order to determine whether there was a corresponding effect on protein expression, Transwell inserts containing THP-1 were transferred to fresh culture media for an additional 16 h (24 h from the initiation of co-culture), then supernatant was collected and assayed for VEGFA protein expression. Data show that VEGFA levels in culture media increased 8- to 10-fold in THP-1 macrophages after co-culture with adipocytes when compared to macrophages cultured alone (Figure 2B).

In order to determine if the effect was species or cell-type specific, we set up a similar co-culture system using murine 3T3-L1 cells differentiated into adipocytes, and murine J774.A1 macrophages (Figure 2C). Here, we also observed a 3- to 4-fold increase in VEGFA mRNA expression by J774.A1 macrophages when exposed to 3T3-L1 adipocytes for 8 h (Figure 2D). We also determined the expression of VEGF protein by the murine macrophages after an additional 16 h, as described above. Again, we observed a robust increase in macrophage VEGFA protein secretion after an 8 h co-culture with adipocytes. This was similar to what was observed for the human co-culture system, except that murine macrophages were not seen to produce any detectable VEGFA protein if they were not exposed to adipocytes for 8 h (Figure 2E).

**Figure 3:**
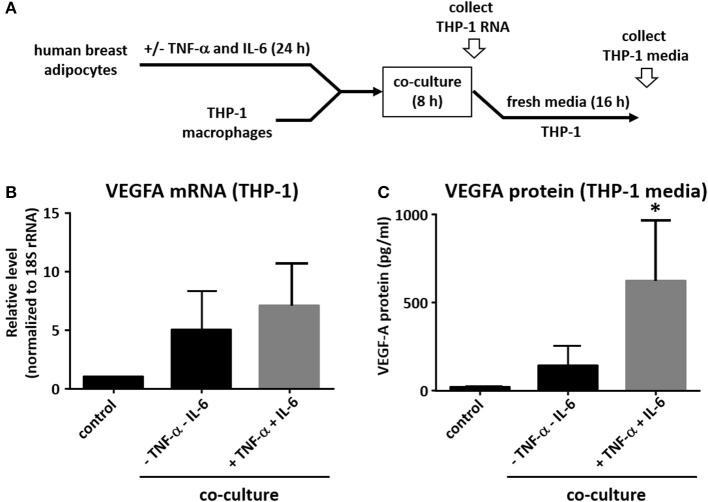
Pre-treatment of adipocytes with IL-6 and TNF-α potentiates effect on macrophage VEGFA expression. **(A)** Differentiated primary human breast adipocytes were treated with TNF-α and IL-6 (10ng/mL each) for 24 h before co-culture with PMA-treated THP-1 using a Transwell system for 8 h. **(B)** Macrophage RNA was collected, reverse transcribed, and qPCR performed with 18S and VEGFA primers. **(C)** Macrophages were co-cultured with pre-treated adipocytes as above for 8 h then changed to new media for 16 additional h before supernatant was collected and analyzed for secreted VEGFA protein (*n* = 3, ^*^p < 0.05 as compared to co-culture without pre-treatment); statistical analyses were performed using standard deviation and the unpaired Student's *t*-test.

Adipocytes are capable of secreting pro-inflammatory cytokines, including CCL2, TNF-α, and IL-6 and their production is significantly increased in obesity (29). In order to model the pro-inflammatory environment in which adipocytes exist in obese adipose tissue, we pre-treated the human breast adipocytes with IL-6 and TNF-α for 24 h before repeating co-culture experiments as described above (Figure 3A). Although the effect on VEGFA mRNA was modest (Figure 3B), we observed a robust increase in macrophage VEGFA protein levels when results were compared to co-culture experiments performed without cytokine pretreatment of adipocytes (Figure 3C).

### VEGFA Expression in Macrophages Treated With Potential Adipocyte-Derived Mediators

**Figure 4:**
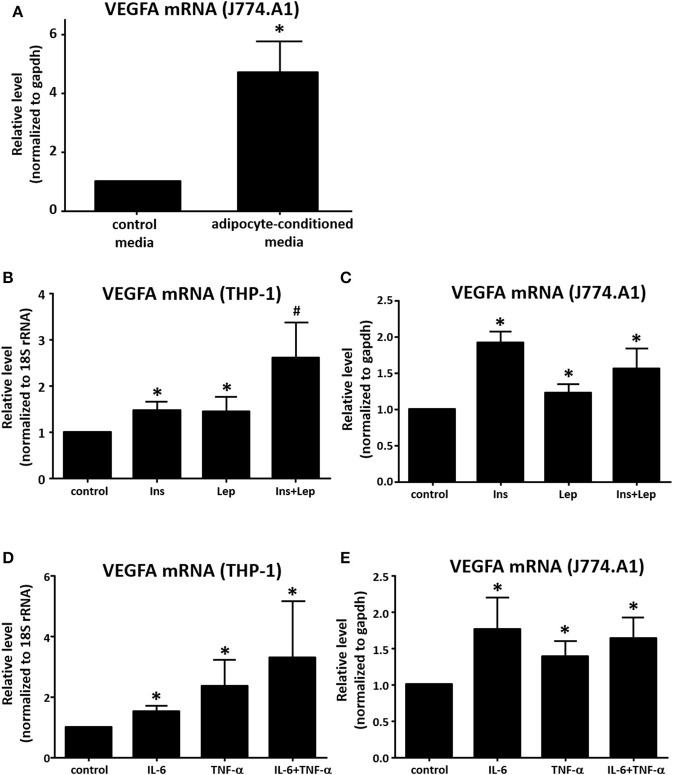
Determination of effects of adipocyte mediators on macrophage VEGF expression. **(A)** Murine 3T3-L1 were differentiated for 12 days, media was changed to fresh DMEM + insulin and cells were allowed to incubate for an additional 48 h. Media was then collected and placed onto murine J774A.1 macrophages plated 24 h prior. After 8 h of treatment with the adipocyte-conditioned media [or fresh DMEM + insulin (non-conditioned) as a control] RNA was collected, reverse transcribed and analyzed for transcriptional changes via qPCR using GAPDH and VEGFA primers (*n* = 3, ^*^p < 0.05). **(B)** PMA-differentiated THP-1 macrophages were treated for 8 h with insulin (500 nM), leptin (1 μg/mL), or insulin (500 nM) + leptin (1 μg/mL), then RNA was collected and analyzed by qPCR (*n* = 4, ^*^p < 0.05) statistical analyses were performed using standard deviation and the unpaired Student's *t*-test. **(C)** J774A.1 macrophages were treated for 8 h with insulin (500 nM), leptin (1 μg/mL), or insulin (500 nM) + leptin (1 μg/mL), then RNA was collected and analyzed by qPCR (*n* = 3, ^*^p < 0.05); statistical analyses were performed using standard deviation and the unpaired Student's *t*-test. **(D)** PMA-differentiated THP-1 macrophages were treated for 8 h with IL-6 (10 ng/mL), TNF-α (10 ng/mL), or IL-6 (10 ng/mL) + TNF-α (10 ng/mL), then RNA was collected and analyzed by qPCR (*n* = 4, ^*^p < 0.05); statistical analyses were performed using standard deviation and the unpaired Student's *t*-test. **(E)** J774A.1 macrophages were treated for 8 h with IL-6 (10 ng/mL), TNF-α (10 ng/mL), or IL-6 (10 ng/mL) + TNF-α (10 ng/mL), then RNA was collected and analyzed by qPCR (*n* = 3, ^*^p < 0.05); statistical analyses were performed using standard deviation and the unpaired Student's *t*-test.

In addition to co-culture, we also performed a media transfer experiment. DMEM media was collected from murine adipocytes after 48 h, transferred to murine J774A.1 macrophages for 8 h, then RNA was collected and assayed for VEGFA expression by real time qPCR. We found the adipocyte-conditioned media stimulated macrophage VEGFA expression in a similar manner to co-culture (Figure 4A). We then assayed candidate mediators from adipocytes for their effect on VEGFA expression in macrophages. Leptin has been shown to up-regulate VEGFA expression in mouse mammary tumor cell lines (30). Insulin is also known to increase VEGFA expression in human retinal pigment epithelial cells (31). Therefore, we treated differentiated THP-1 human and J774A.1 murine macrophages with insulin, leptin, or the combination of both, then assessed the effect on VEGFA mRNA expression. In both the human and the murine cells, we observed increases in macrophage VEGFA production with insulin or leptin alone (Figures 4B,C). In THP-1 macrophages we found that insulin and leptin in combination had a greater effect than either alone, however this effect was not observed in murine macrophages. Several studies have shown that IL-6 enhances VEGFA levels in various cell cancer types including gastric carcinoma, osteosarcoma, and multiple myeloma (32–34). The pro-inflammatory cytokine TNF-α has also been reported to up-regulate expression of VEGFA (35). Therefore, we treated THP-1 and J774.A1 macrophages with IL-6 alone, TNF-α alone, or the combination of both cytokines and assessed expression of VEGFA mRNA. Our data show that either IL-6 or TNF-α were capable of enhancing VEGFA expression in THP-1 and J774A.1 macrophages (Figures 4D,E), but the combination of IL-6 + TNF-α did not increase VEGFA expression beyond what was observed with either agent alone. We also tested the adipocyte signaling protein, visfatin to determine whether it was capable of enhancing VEGFA mRNA expression in THP-1 macrophages, but we did not observe a significant effect (data not shown).

### Macrophage Production of Angiogenesis-Related Proteins Is Increased by Exposure to Adipocytes

**Figure 5:**
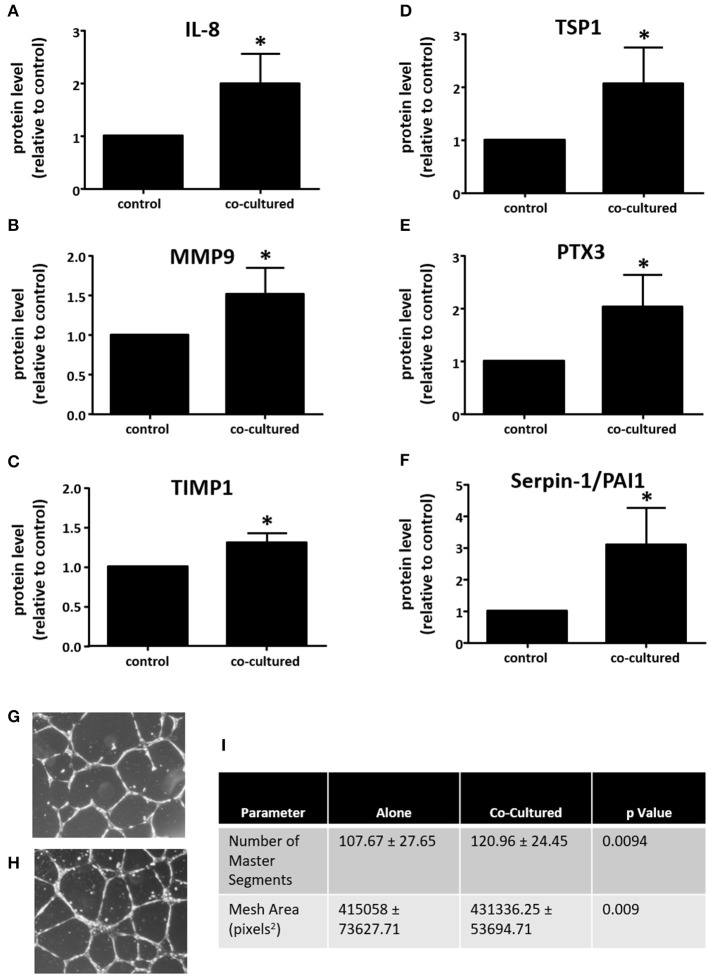
Adipocyte-exposed macrophages produce more angiogenesis regulating proteins. THP-1 were differentiated with PMA and either cultured alone (control) or with primary human breast adipocytes for 8 h, then changed to fresh media for an additional 16 h before collecting conditioned media, which was assayed on Angiogenesis Array nitrocellulose membranes pre-spotted with capture antibodies for 55 angiogenesis related proteins (*n* = 3, ^*^p < 0.05); statistical analyses were performed using standard deviation and the unpaired Student's *t*-test. **(A)** IL-8; **(B)** MMP9; **(C)** TIMP1; **(D)** TSP1; **(E)** PTX3; and, **(F)** Serpin-E1/PAI1 protein expression levels. Endothelial tube formation assay was performed with adipocyte-exposed macrophage supernatant **(H)** and control macrophage supernatant **(G)**. HUVEC incubated in media conditioned with macrophages not exposed to adipocytes. **(I)** Table of parameters measured that significantly increased in the HUVEC tube formation assay when HUVEC were incubated in media conditioned with macrophages cultured alone (control) or those co-cultured with adipocytes [statistical analyses were performed using standard deviation and the 2-way ANOVA with factors for experiment (*n* = 4) and treatment (macrophages cultured alone vs. macrophages co-cultured with adipocytes)].

Since co-culture with adipocytes increased macrophage expression of VEGFA, we wanted to determine whether co-culture influenced other macrophage proteins that could regulate angiogenesis. For this purpose, we used the R&D Systems Proteome Profiler Human Angiogenesis Array to assay media from adipocyte-exposed macrophages, comparing these data to media from macrophages cultured alone. Here, we observed significant increases in the expression of several pro-angiogenic and pro-metastatic genes, including IL-8, MMP9, PTX3, and Serpin E1/PAI1. While the majority of changes were in angiogenesis-promoting proteins, there were two proteins with increased expression that are associated with inhibition of angiogenesis TIMP1, and TSP1 (Figures 5A–F).

### Supernatant From Adipocyte-Exposed Macrophages Induces Endothelial Tube Formation

Since our data show that co-culture with adipocytes or treatment with adipocyte-conditioned media enhanced the expression of VEGFA and other mediators of angiogenesis in macrophages, we next performed an endothelial tube formation assay with HUVEC to determine whether there was a functional effect on an *in vitro* model of angiogenesis. THP-1 macrophages were co-cultured with human breast adipocytes for 8 h as described above, then transferred to fresh media and cultured an additional 16 h. This media was collected and then used to evaluate angiogenic activity. As a control we cultured macrophages on their own for 8 h, then transferred them to fresh media for 16 h. As we would anticipate with the inclusion of fetal bovine serum, both supernatants stimulated endothelial cell tube formation. However, HUVEC treated with media from adipocyte-exposed macrophages (Figure 5H) formed more complex network of tubules than HUVEC treated with media from macrophages cultured alone (Figure 5G). We quantified parameters of tube formation using the Angiogenesis Analyzer tool developed by Gilles Carpentier for ImageJ image analysis software. In the Angiogenesis Analyzer tool, Segments refer to lines delimited by two Junctions, which are points where two or more Segments join. Master Segments are Junctions linking at least three Master Segments, which are Segments delimited by two Master Junctions. A Mesh is an area completely enclosed by Segments (22). The number of Master Segments and the Mesh Area both increased when HUVEC were incubated with conditioned media from macrophages co-cultured with adipocytes, as compared to HUVEC incubated with media from macrophages cultured alone (Figure 5I).

**Figure 6:**
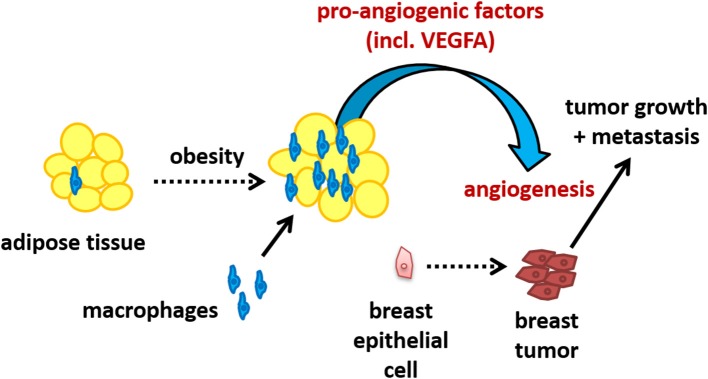
Schematic illustrating the potential of the adipocyte-macrophage interaction in obese breast adipose tissue to promote angiogenesis and thus metastasis.

In summary, we have found that incubation of macrophages with human breast adipocytes increases the production of VEGFA as well as other pro-angiogenic mediators. The functional effect of this interaction was demonstrated by an enhancement in endothelial cell tube formation, suggesting an increase in metastatic potential due to the adipocyte-macrophage interaction. We propose that the recruitment of macrophages into obese breast tissue results in increased production of pro-angiogenic signaling, particularly VEGFA, which could promote tumor vascularization and represents a mechanism whereby obesity enhances the risk of breast cancer metastasis. This proposed pathway is illustrated in Figure 6.

## Discussion

Epidemiological studies have linked obesity with an increase in the growth and dissemination of breast cancer (36). We have investigated the adipocyte-macrophage interaction as a mechanism by which obesity could influence macrophage function to promote angiogenesis and metastasis of breast tumors.

To study the interaction of adipocytes and macrophages, we used a Transwell co-culture system to explore whether co-culture with human breast adipocytes influenced macrophage phenotypes. Macrophages can be classified as either an M1 or M2 phenotype, which can be distinguished by whether they synthesize nitric oxide (M1) or polyamines (M2), among other differences in gene expression patterns (23, 26, 37). Rather than having a distinct M1 or M2 phenotype, co-cultured macrophages exhibited a mixed M1/M2 phenotype, which is in agreement with earlier work examining the phenotype of adipose tissue macrophages. Accordingly, macrophages isolated from subcutaneous and visceral human adipose tissue have been shown to express M1 pro-inflammatory cytokines including IL-6 and TNF-α, as well as significant levels of M2 markers including IL-10 and MRC1 (38). Similarly, macrophages isolated from human adipose tissue have been found to display a phenotype which is unique from monocyte-derived macrophages, and which is capable of altering gene expression in human cancer cell lines (39). A recent study showed infiltration of macrophages of mixed polarity in the adipose tissue adjacent to breast tumors and that high levels of macrophages correlated with a higher histologic grade (40).

With this in mind, we next investigated the effects of co-culture on macrophage gene expression relating to angiogenesis and metastasis. When either human or murine macrophages were exposed to adipocytes, the expression of VEGFA and other pro-angiogenic factors was significantly increased. Furthermore, we found that supernatant from the adipocyte-exposed macrophages was capable of promoting endothelial tube formation in an *in vitro* angiogenesis assay to a greater extent than macrophages cultured alone. In invasive breast carcinomas, TAMs have been shown to express a significant level of VEGFA mRNA and protein, suggesting a role for macrophages in tumor angiogenesis (41). Additional work has shown that VEGFA expression is enhanced by co-culture of mouse peritoneal macrophages with adipocytes and mammary tumor cells, but did not explore the specific influence of adipocytes on macrophage phenotype and macrophage-derived VEGFA levels (42). Here, we have isolated secreted proteins from macrophages only, after prior exposure to adipocytes, in order to determine the source of origin of the increased VEGFA. Our analyses demonstrate that the macrophage VEGFA secretion was enhanced through an interaction with the adipocytes alone.

Previous studies have shown a relationship between adipose tissue and tumor angiogenesis *in vivo*. B16F10 melanoma or Lewis lung carcinoma cells implanted within or near subcutaneous adipose tissue deposits in mice grew more rapidly and were more vascularized than tumors implanted in non-adipose tissue locations (43). A humanized murine breast cancer model was used to demonstrate that adipose tissue and the presence of macrophages within the mammary tissue promote stromal vascularization and angiogenesis (44). In a murine mammary tumor model depletion of macrophages in obese mice led to a reduction in CD31^+^ endothelial cells whereas macrophage depletion did not impact tumor angiogenesis in lean mice (45). This would suggest that macrophages are a key component in promotion of angiogenesis by obese adipose tissue. Our study adds to these findings by demonstrating that the interaction between the adipocytes and macrophages in the absence of tumor cells is sufficient to promote a pro-angiogenic phenotype in the macrophages, particularly the expression of VEGFA. Furthermore, this is the first study to specifically culture human breast adipocytes showing the relevance of this interaction in the context of obese human breast adipose tissue.

We have also explored which adipocyte-derived mediators are capable of stimulating the expression of VEGFA in macrophages. We found that insulin, leptin, IL-6 and TNF-α, which are each known to be produced by adipocytes, induced the expression of VEGFA by macrophages. We suspect that a combination of soluble adipocyte-derived signals is involved in up-regulating macrophage expression of VEGFA. Prior studies have demonstrated that these adipocyte mediators can alter gene expression in macrophages. For example, leptin has been shown to stimulate the expression of pro-inflammatory cytokines in human mononuclear cells, and to up-regulate toll-like receptor 2 (TLR2) expression in THP-1 macrophages (46, 47). Treatment of MCF-7 and MDA-MB-231 breast cancer cells with leptin led to increased expression of VEGFA (48). Here we show that leptin can also induce VEGFA expression in macrophages, suggesting that adipocyte-derived signals may promote tumorigenesis by influencing the tumor microenvironment, as well as the tumor cells directly.

One recent study profiled the expression of pro-inflammatory cytokines in visceral and subcutaneous adipose tissue derived from patients undergoing bariatric surgery. The authors demonstrated a positive correlation between the expression of CCL2, which acts as a chemoattractant for macrophages, and the expression of IL-6 and TNF-α. They also found a positive correlation between TNF-α expression and the expression of VEGFA (49). These data support our findings, in which macrophages, pro-inflammatory cytokine expression, and VEGFA expression are enhanced within adipose tissue that is marked with macrophage infiltration.

Obesity has been linked with breast cancer metastasis, but the mechanisms behind this correlation have not yet been fully resolved. Macrophages are abundant in the breast tissue of obese women, compared to non-obese breast tissue. Adipocytes can signal to and influence the phenotype of the macrophages. We have demonstrated that VEGFA is up-regulated in macrophages after exposure to adipocytes and that the supernatant from adipocyte-exposed macrophages can stimulate endothelial tube formation. This effect could be mediated through the release of soluble adipocyte mediators such as insulin, leptin, TNF-α, and IL-6. We have demonstrated this effect with human breast adipocytes in co-culture with macrophages, a system that has not previously been studied *in vitro*. Our data provide mechanistic insight to previous *in vivo* studies which have demonstrated increased tumor angiogenesis in the presence of adipose tissue and outlines a possible means by which obesity can promote breast cancer growth and metastasis.

## Data Availability Statement

All datasets generated for this study are included in the article/supplementary material.

## Author Contributions

NY assisted with the study design, performed experiments, analyzed and interpreted data, and assisted with drafting the manuscript. AB and YU assisted in performing experiments. LC and AJ conceptualized and designed the study, analyzed and interpreted data, and assisted in drafting the manuscript. All authors read and approved the manuscript.

### Conflict of Interest

The authors declare that the research was conducted in the absence of any commercial or financial relationships that could be construed as a potential conflict of interest.
